# Draft genome sequences of two *Enterococcus faecium* strains recovered from blood cultures

**DOI:** 10.1128/mra.00683-26

**Published:** 2026-09-08

**Authors:** Wenjing Wen, Ning Sun, Yongchao Ma

**Affiliations:** 1 Department of Pathogen Biology and Immunology, Luohe Medical College 338952, Luohe, Henan, People’s Republic of China; 2 Department of Clinical Laboratory, Jinling Hospital, Affiliated Hospital of Medical School, Nanjing University 12581, Nanjing, China; 3 Department of Biochemistry, Luohe Medical College 338952, Luohe, Henan, People’s Republic of China; University of Pittsburgh School of Medicine, Pittsburgh, Pennsylvania, USA

**Keywords:** *Enterococcus faecium*, plasmids, genomes

## Abstract

We report the draft genomes of two *Enterococcus faecium* blood isolates: one vancomycin-susceptible and one vancomycin-resistant. The assemblies comprised 2,991,512 bp (7 contigs) for the susceptible strain and 3,305,414 bp (7 contigs) for the resistant strain.

## ANNOUNCEMENT

*Enterococcus faecium* colonizes the human gut and causes bloodstream infections, especially in immunocompromised patients (1). High resistance rates to ampicillin and ciprofloxacin leave glycopeptides like vancomycin as a crucial option for severe infections (1). This species also carries diverse virulence genes involved in biofilm formation, nutrient acquisition, and toxin production (1, 2), and plasmids play a key role in spreading resistance and virulence genes by horizontal gene transfer (3). We report draft genome sequences of two clinical *E. faecium* isolates (efmnj01 and efmnj02) from blood cultures collected at Jinling Hospital, Nanjing, China.

Strains were cultured on blood agar (Kemajia, Shanghai, China) overnight at 35°C with 5% CO₂, and a single colony was transferred to Brain Heart Infusion Broth (Sangon, Shanghai, China). Genomic DNA was extracted using the TaKaRa MiniBEST Kit (Takara, Dalian, China). Illumina sequencing libraries were prepared with Rapid Plus DNA Lib Prep Kit for Illumina (ABclonal, Wuhan, China) from sheared DNA (~350 bp) and sequenced as paired-end (2 × 150  bp) on a NovaSeq X + platform, generating 15.8 million reads for efmnj01 and 12.6 million reads for efmnj02. Reads were quality-filtered with fastp (v1.3.6) (4). Nanopore libraries were prepared with the Native Barcoding Kit 96 V14 (SQK-NBD114.96, Oxford Nanopore Technologies), pooled, and sequenced on a PromethION flow cell R10.4.1 (FLO-PRO114M). Base calling was performed with MinKNOW (v23.07.5) using the high-accuracy model. Nanopore sequencing generated 1.50 Gb (263,680 reads; N50 = 17.2 kb) for efmnj01 and 1.44 Gb (149,304 reads; N50 = 19.2 kb) for efmnj02. We filtered the long reads with Filtlong (v0.3.1, --min_length 1000—keep_percent 95) and assembled with Flye (v2.9.6-b1802) (5). Flye automatically trimmed the overlapping terminal repeats of the circular contigs; genomes were not rotated. Assemblies were polished with two rounds of Pilon (v1.24) (6) using Illumina reads aligned with BWA-MEM (v0.7.17) (7). Average sequencing depths were 459× for efmnj01 and 406× for efmnj02. Sequence types were determined *in silico* using the *E. faecium* MLST scheme. Annotation was performed with the Prokaryotic Genome Annotation Pipeline (PGAP) (v6.11) (8–10). Plasmid, virulence, and resistance genes were identified using PlasmidFinder (v2.1.6) (11), VirulenceFinder (v2.0.4) (12), and ResFinder (v4.6.0) (13), respectively. All tools were run with default parameters unless otherwise specified.

Assembly statistics are provided in Table 1. The vancomycin-susceptible ST78 strain efmnj01 has a 2,991,512-bp genome (GC 37.95%) in 7 contigs (N50 = 2,729,572 bp) and carries three circular plasmids (228, 16.5, and 13.5 kb) with replicons repUS15, rep29, and 1 unknown. PGAP predicted 2,854 coding sequences (CDS), 69 transfer RNA (tRNA), and 20 ribosomal RNA (rRNA) genes. The vancomycin-resistant ST363 strain efmnj02 has a 3,305,414-bp genome (GC 37.61%) in 7 contigs (N50 = 2,837,821 bp) and carries 5 circular plasmids (163, 58, 58, 40.6, and 11.9 kb) and 1 linear plasmid (135.6 kb). The 58-kb plasmid (contig_5) harbors *vanHAX*, *erm(B*), *tet(M*), *tet(L*), *cat*, and *aph(3′)-III*. PGAP predicted 3,220 CDS, 72 tRNA, and 18 rRNA genes. These two draft genomes illustrate the genomic and plasmid diversity of *E. faecium* and highlight the role of plasmids in disseminating resistance and virulence determinants among clinical isolates.

**TABLE 1:** Statistics of the genome assemblies^*a*^

Strain	Contig	Length (bp)	GC (%)	Type	Topology	Repeat	Mult.	Replicon types	AMR genes	Accession number
efmnj01	contig_1	2,729,572	38.2	Chromosome	Circular	N	1	repUS12, repUS43 (chromosomal)	*aac(6′)-Ii*, *msr(C), erm(T), tet(M), tet(L), dfrG*	JBYRUW000000000
efmnj01	contig_7	227,928	35.3	Plasmid	Circular	N	1	repUS15	*aac(6′)-aph(2*″), *aph(2″)-Ia*	
efmnj01	contig_2	16,450	33.9	Plasmid	Circular	Y	1	unknown	–	
efmnj01	contig_9	13,542	34.5	Plasmid	Circular	Y	2	rep29	–	
efmnj01	contig_8	2,604	42.9	Unplaced contig	Linear	Y	4	–	–	
efmnj01	contig_5	732	40.2	Unplaced contig	Linear	Y	4	–	–	
efmnj01	contig_3	684	35.5	Unplaced contig	Linear	Y	2	–	–	
efmnj02	contig_1	2,837,821	38.0	Chromosome	Circular	N	1	repUS12, repUS43 (chromosomal)	*aph(2″)-Ie*, *aac(6′)-Ii*, *msr(C*), *tet(M*), *tet(L*), *dfrG*	JBYTSQ000000000
efmnj02	contig_6	163,244	35.2	Plasmid	Circular	N	1	repUS15	–	
efmnj02	contig_2	135,571	35.0	Plasmid	Linear	N	1	none detected	–	
efmnj02	contig_4	58,230	35.9	Plasmid	Circular	N	1	rep17	–	
efmnj02	contig_5	58,071	35.8	Plasmid	Circular	Y	3	repUS47, Inc18 (rep2)	*aph(3′)-III*, *erm(B*), *cat*, *tet(M*), *tet(L*), *VanHAX*	
efmnj02	contig_8	40,598	32.6	Plasmid	Circular	N	1	none detected	–	
efmnj02	contig_7	11,877	33.5	Plasmid	Circular	Y	8	rep18b	–	

^*a*^ “Unplaced contig” refers to short, linear fragments with high repeat content and multi‑copy numbers, likely originating from assembly artifacts or fragmented repeats; “Repeat” (Y/N) indicates whether the contig was flagged as a repetitive region in the Flye assembly graph; “Mult.” indicates the copy number of a contig in the assembly graph; “Replicon types” were determined using PlasmidFinder (v2.1.6); “AMR gene” (antimicrobial resistance genes) were identified using ResFinder (v4.6.0); “–” indicates that the corresponding feature was not detected. Both analyses were performed with default parameters.

## Data Availability

This whole-genome shotgun project has been deposited in GenBank under BioProject accession number PRJNA1469365. The BioSample accession numbers for strains efmnj01 and efmnj02 are SAMN60354743 and SAMN60354744, respectively. The genome assemblies have been assigned the GenBank accession numbers JBYRUW000000000 (efmnj01) and JBYTSQ000000000 (efmnj02). The raw sequencing reads have been deposited in the Sequence Read Archive under the accession numbers SRR38795422 (efmnj01) and SRR38795421 (efmnj02). The version described in this paper is the first version.

## References

[1] Wei Y, Palacios Araya D, Palmer KL. 2024. *Enterococcus faecium*: evolution, adaptation, pathogenesis and emerging therapeutics. Nat Rev Microbiol 22:705–721. doi:10.1038/s41579-024-01058-6 PMC12147861 38890478

[2] Lu Z, Mclnnes RS, Allen F, Gadar K, van Schaik W. 2025. Resistance to last-resort antibiotics in enterococci. FEMS Microbiol Rev 49:fuaf057. doi:10.1093/femsre/fuaf057 PMC12641126 41206754

[3] Lang AS, Buchan A, Burrus V. 2025. Interactions and evolutionary relationships among bacterial mobile genetic elements. Nat Rev Microbiol 23:423–438. doi:10.1038/s41579-025-01157-y 40069292

[4] Chen S, Zhou Y, Chen Y, Gu J. 2018. Fastp: an ultra-fast all-in-one FASTQ preprocessor. Bioinformatics 34:i884–i890. doi:10.1093/bioinformatics/bty560 PMC6129281 30423086

[5] Kolmogorov M, Yuan J, Lin Y, Pevzner PA. 2019. Assembly of long, error-prone reads using repeat graphs. Nat Biotechnol 37:540–546. doi:10.1038/s41587-019-0072-8 30936562

[6] Walker BJ, Abeel T, Shea T, Priest M, Abouelliel A, Sakthikumar S, Cuomo CA, Zeng Q, Wortman J, Young SK, Earl AM. 2014. Pilon: an integrated tool for comprehensive microbial variant detection and genome assembly improvement. PLoS One 9:e112963. doi:10.1371/journal.pone.0112963 PMC4237348 25409509

[7] Li H. 2013. Aligning sequence reads, clone sequences and assembly contigs with BWA-MEM. arxiv. doi:10.48550/arXiv.1303.3997

[8] Tatusova T, DiCuccio M, Badretdin A, Chetvernin V, Nawrocki EP, Zaslavsky L, Lomsadze A, Pruitt KD, Borodovsky M, Ostell J. 2016. NCBI prokaryotic genome annotation pipeline. Nucleic Acids Res 44:6614–6624. doi:10.1093/nar/gkw569 PMC5001611 27342282

[9] Haft DH, DiCuccio M, Badretdin A, Brover V, Chetvernin V, O’Neill K, Li W, Chitsaz F, Derbyshire MK, Gonzales NR, Gwadz M, Lu F, Marchler GH, Song JS, Thanki N, Yamashita RA, Zheng C, Thibaud-Nissen F, Geer LY, Marchler-Bauer A, Pruitt KD. 2018. RefSeq: an update on prokaryotic genome annotation and curation. Nucleic Acids Res 46:D851–D860. doi:10.1093/nar/gkx1068 PMC5753331 29112715

[10] Li W, O’Neill KR, Haft DH, DiCuccio M, Chetvernin V, Badretdin A, Coulouris G, Chitsaz F, Derbyshire MK, Durkin AS, Gonzales NR, Gwadz M, Lanczycki CJ, Song JS, Thanki N, Wang J, Yamashita RA, Yang M, Zheng C, Marchler-Bauer A, Thibaud-Nissen F. 2021. RefSeq: expanding the prokaryotic genome annotation pipeline reach with protein family model curation. Nucleic Acids Res 49:D1020–D1028. doi:10.1093/nar/gkaa1105 PMC7779008 33270901

[11] Carattoli A, Zankari E, García-Fernández A, Voldby Larsen M, Lund O, Villa L, Møller Aarestrup F, Hasman H. 2014. *In silico* detection and typing of plasmids using PlasmidFinder and plasmid multilocus sequence typing. Antimicrob Agents Chemother 58:3895–3903. doi:10.1128/AAC.02412-14 PMC4068535 24777092

[12] Joensen KG, Scheutz F, Lund O, Hasman H, Kaas RS, Nielsen EM, Aarestrup FM. 2014. Real-time whole-genome sequencing for routine typing, surveillance, and outbreak detection of verotoxigenic *Escherichia coli**.* J Clin Microbiol 52:1501–1510. doi:10.1128/JCM.03617-13 PMC3993690 24574290

[13] Bortolaia V, Kaas RS, Ruppe E, Roberts MC, Schwarz S, Cattoir V, Philippon A, Allesoe RL, Rebelo AR, Florensa AF, et al.. 2020. ResFinder 4.0 for predictions of phenotypes from genotypes. J Antimicrob Chemother 75:3491–3500. doi:10.1093/jac/dkaa345 PMC7662176 32780112

